#  Classification of G-Quadruplex DNA on the Basis of the Quadruplex Twist Angle and Planarity of G-Quartets 

**Published:** 2010

**Authors:** R.V. Reshetnikov, A.M. Kopylov, A.V. Golovin

**Affiliations:** Faculty of Bioengineering and Bioinformatics, Lomonosov Moscow State University; Faculty of Chemistry, Lomonosov Moscow State University; Belozersky Institute of Physico-Chemical Biology, Lomonosov Moscow State University; Apto-Pharm LLC

**Keywords:** G-quadruplex, G-quartet, twist angle, loops, structure

## Abstract

The present work is devoted to the analysis of the G-quadruplex DNA structure using the bioinformatics method. The interest towards quadruplex DNAs is determined by their involvement in the functioning of telomeres and onco-promoters as well as by the possibility to create on their basis aptamers and nanostructures. Here, we present an algorithm for a general analysis of the polymorphism of the G-quadruplex structure from the data bank PDB using original parameters. 74 structures were grouped according to the following parameters: the number of DNA strands, the number of G-quartets, and the location and orientation of the connecting loops. Two quantitative parameters were used to describe the quadruplex structure: the twist angle between two adjacent quartets (analogous to that for the complementary pair in the duplex DNA) and the quartet planarity (an original parameter). The distribution patterns of these values are specific for each group of quadruplex structures and are dependent upon the type of connecting loops used (diagonal, lateral or propeller). The tetramolecular loopless parallel quadruplex was used as a comparison template. The lateral loops introduce the strongest distortion into the structure of quadruplexes: the values of the twist angles are the lowest and are not typical for the other quadruplex groups. The loops of the diagonal type introduce much weaker deformation into quadruplexes; the structures with propeller loops are characterized by the optimum geometry of G-quartets. Hence, the correlation between the twist angle and the tension in the structure of quadruplex DNA is revealed.

##  INTRODUCTION 

**G-quadruplexes**

**Fig. 1:**
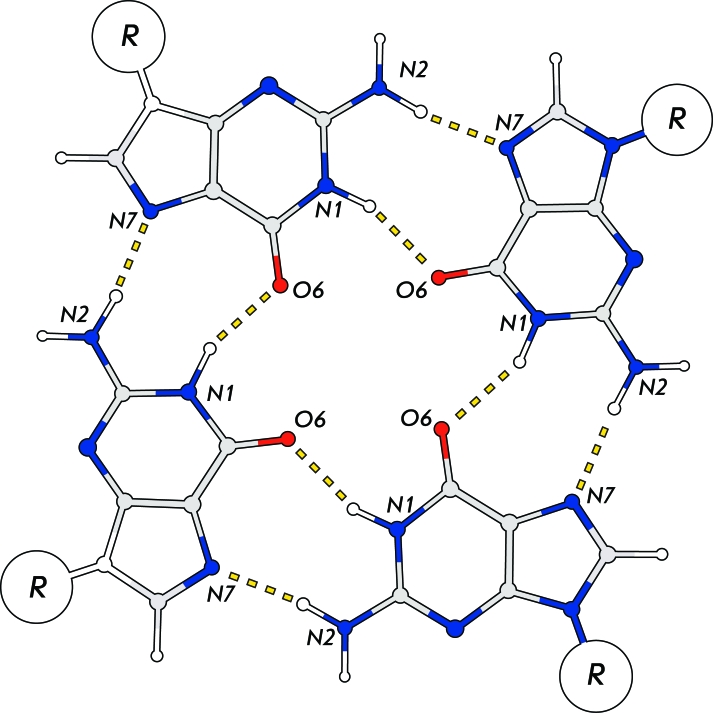
Scheme of G-quartet. Four guanine residues form a square coplanar structure, where each nucleic acid base is a donor and acceptor of a hydrogen bond: N1 and N2 from one side of the heterocycle, O6 and N7 from the other side of the guanosine involved in formation of 8 hydrogen bonds in one quartet. Sugar-phosphate backbone of nucleic acids is denoted as R.

It is known that the strands of guanosine oligo- and polynucleotides are capable of aggregating with each other, on condition that the solution contains a monovalent cation, such as potassium or sodium. X-ray diffraction analysis revealed that these poly(G)-strands represent a novel type of DNA folding, a four-strand helix [1–3], where four guanine bases belonging to different strands form a planar structure retained by G-G pair interactions (Fig. 1). These structures are notable for their high stability and are known as guanine (G)-quartets, or G-tetrads. Each G-quartet is bound by a total of eight hydrogen bonds, which are formed as a result of the interaction between the Watson-Crick side of one guanine base and the Hoogsteen side of another one.

One of the fundamental reasons the nucleic acids containing the G-tetrad motif are of interest is that the guanine-rich sequences are widely represented in all the genomes that have been discovered to date. These motifs were found in the promoter regions and immunoglobulin switch regions, recombination hotspots, etc. [4]. G-quartets are also represented in DNA at the ends of eucariotic chromosomes known as telomeres [5]. Telomeric DNAs are tandem repeats of short poly-G-blocks, sometimes comprising adenine or thymine bases: G _n_ T _n_ , G _n_ T _n_ G _n_ , G _n_ A _n_ or (TTAGGG) _n_ ; telomeric DNAs are associated with telomeric proteins. The repeat types are species-dependent; for instance, the (TTAGGG) _n_ repeat is typical of mammals. The function of the telomeres is to protect the chromosomal ends from damage under recombination or action of nucleases. Human telomeric DNA in somatic cells contains, on average, 8–10 kbp. The terminal 100–200 nucleotides at the 3’-end represent a conformationally unrestricted single-strand “tail” [6]. In living cells, the “tail” is associated with POT1 protein [7]; while in the absence of this protein the single-stranded DNA is capable of folding and dimerizing to form four-stranded hairpins, which can be stabilized by the formation of guanine tetrads [8, 9]. An alternative method for the stabilization of this type of DNA consists in the formation of intramolecular G-quartets by repeated foldback. These G-quartet-containing structures are known as quadruplexes or tetraplexes [10]. Aside from telomeres, G-quadruplex sequences were localized in promoters of a number of oncogenes and cancer-associated genes, such as *k-ras * [11], *c-kit* [12], and  *bcl2 * [13]. Thus, the possibility of inhibition of the corresponding gene expression using such quadruplex-specific agents as porphyrin TMPyP4 [14, 15] appears to be encouraging.

G-quadruplexes can also be formed by short oligonucleotides with the corresponding sequence, which can be written as G _m_ X _n_ G _m_ X _o_ G _m_ X _p_ G _m_ , where m is the number of guanine per G-block. These guanines are generally directly involved in G-tetrad formation. X _n_ , X _o_ and X _p _ can be a combination of any residues, including G; these regions form loops between the G-tetrads.

**Fig. 2:**
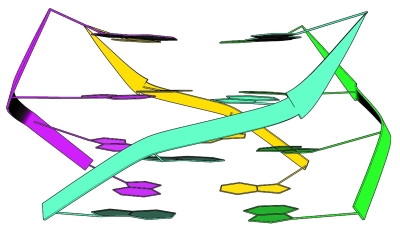
Four-stranded intermolecular parallel G-quadruplex.

Some of these sequences exhibit aptameric properties upon folding into quadruplex structures. Aptamers are short synthetic oligonucleotides or peptides, being functional analogs of monoclonal antibodies, and are capable of specifically recognizing a wide range of targets, from small molecules to whole cells [16, 17]. G-quadruplex aptamers targeting a wide range of proteins, such as thrombin [18] and STAT-3 [19], have been identified. There are G-quadruplex aptamers with anti-cancer properties, which are currently undergoing clinical trials. Their mechanism of action is connected with the nucleolin protein and its role in RNA processing [20].

**Common structural features of quadruplex DNAs**

**Fig. 3:**
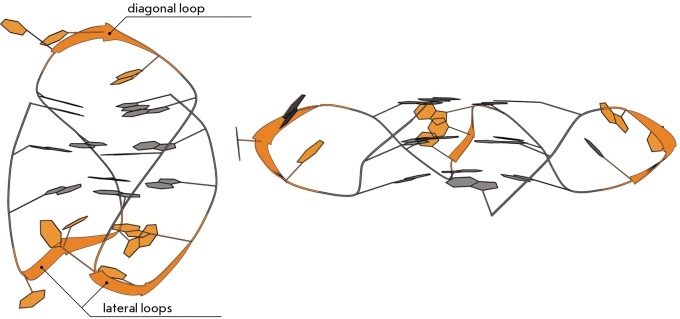
Folding of a DNA strand into a monomolecular G-quadruplex with three G-quartets. Two variants of oligonucleotide d(AGGG(TTAGGG) _3_ ) differ from each other in the direction of the polynucleotide chain in different strands of the quadruplex. Left: topology with loops of lateral and diagonal type [23]; right: topology with strand-reversal loops of propeller type [24].

 Depending on the number of G-blocks, the formation of a quadruplex structure from a specified quadruplex sequence may occur via different paths. Four individual strands may associate with each other to form an intermolecular G-quadruplex (Fig. 2). Intramolecular tetraplexes are formed from a single-stranded molecule as a result of the complex spatial folding of the nucleotide strand (Fig. 3). 

The G-quartet is a fundamental structural unit of all quadruplex structures. Within the quadruplex structure, the quartets are located one above the other; a minimum of two quartets are required for the structural stability of the tetraplex [21]. The number of guanines in each individual G-block is directly related to the number of G-tetrads in the final folded quadruplex. For example, in telomeric DNA found in mammals with tandem repeat d(TTAGGG), the quadruplexes formed by four of these repeats have three G-quartets located one above the other [22].

The factors that stabilize the quadruplex are the same as those that stabilize the duplex DNA; including the base stacking interaction, hydrogen bonds, electrostatic interactions, and the hydration shell. The hydration of the sugar-phosphate backbone plays a crucial role in the stability of the structure: within an ordered hydration shell, the water molecules with an extensive network of hydrogen bonds combine into a single unit with the bases, sugars, and charged phosphates which are localized on the outer surface of the quadruplex. [25–27].

In addition to these stabilizing factors being standard for the duplex DNA, the quadruplexes have a highly specific component: a significant contribution to stability is made by the coordination of O6 carbonyls by cations [28, 29]. Atoms O6 form a square in each quartet, forming a bipyramidal antiprism in the quadruplex with an interquartet distance of 3.3 Å [30]. This negatively charged space between the tetrads should be stabilized by coordination of a cation. The cation’s nature (size/ionic radius and charge) has a considerable effect on the stability of the resultant quadruplex [29].

Four guanosine nucleosides within a tetrad can exist in either *anti- * or *syn* -conformation with respect to the glicoside bond; thus providing 16 possible combinations. The mutual orientation of the individual strands within a quadruplex has an effect on glicoside angles. For instance, with all four strands oriented in parallel (Fig. 2), all glicoside angles have an *anti* -conformation. For anti-parallel orientation, the quadruplexes contain both *syn* - and *anti* -guanines, regardless of whether the quadruplex is four- or single-stranded.

Various nucleotide sequences between G-blocks form extrahelical loops. These loops may be of three types. The parallel intramolecular quadruplex requires a loop that would connect the bottom G-tetrad with the top one, resulting in the formation of propeller-type loops (Fig. 3, right). Anti-parallel quadruplexes are ones in which at least one strand is located antiparallel to the other strands. Such topology of tetrads was revealed in most of the currently identified bimolecular and intramolecular quadruplex structures. In addition to the propeller-type loops, loops of two more types are observed in these structures. The adjacent G-strands are linked by the lateral (endgewise) loops. Two loops of this type can be located both at the same and opposite poles of the molecule, corresponding to the “head-to-head” or “head-to-tail” arrangement in bimolecular complexes. The second type of anti-parallel loop is represented by the diagonal loops connecting the opposite G-strands (Fig. 3 *, * left).

 All quadruplex structures have four grooves, unlike the double helix with only two grooves. The grooves are formed by the cavities restricted by sugar-phosphate backbones. The groove’s dimensions widely vary depending on the total topology and nature of loops, and on glycoside angles, as well. In the quadruplexes with loops of only diagonal or lateral type, the grooves are structurally simple, while in the structures with propeller-type loops they possess more complex structural features. 

 Thus, there is a large number of structural variables (the number of G-tetrads; type, sequence, length, and orientation of loops), which leads to a wide topological and structural variety of quadruplexes. In this work, we attempted to reveal the interrelation between the structure and properties of G-quadruplex DNAs and determine the factors that have an influence on a quadruplex’s geometry. 

##  METHODS 

**Sampling**

 The PDB data bank was used to compile the list of quadruplex-containing structures. All found structures were divided into 8 groups according to the geometry of spatial organization of a quadruplex. On the basis of the Perl programming language and modules Vector::Real and Statistics::Descriptive, we developed a software tool that would determine the presence of quartets in the structure, reveal their location, and measure their geometrical parameters. The quartet is determined as follows: a guanine should have a neighbor in contact with the O6 atom of the initial guanine via the N7 atom. The combination selected is recognized as a quartet on condition that the fourth guanine interacts with the first one, and all heterocyclic bases are located in one plane with the maximum permissible offset of the atoms from the surface being equal to 2 Å. The nearest quartet from the initial one with the distance between C1’ atoms from the nearest nucleotides being no more than 10 Å was recognized as the next quartet of the quadruplex (thus, the variants with guanines from different quartets forming a tetrad were eliminated). The structures belonging to different NMR models were processed as independent quadruplexes. 

**Fig. 4:**
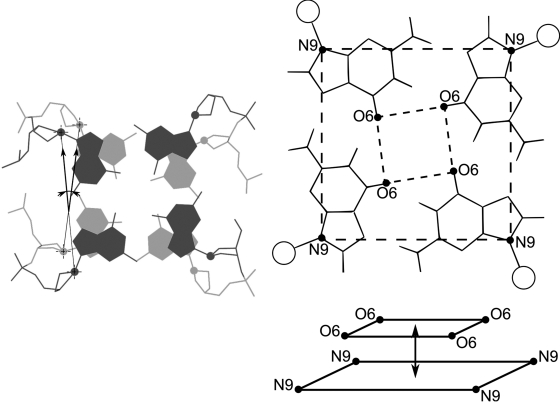
Definition of the measured parameters. Left: Definition of the quadruplex twist angle. C1’ atoms are shown as spheres. Right: relative positions of two tetragons: the outer, formed by N9 atoms, and the inner, formed by O6 atoms. If the planarity of the quartet is disturbed, then the inner tetragon leaves the quartet plane. Distance between the centers of mass of the inner and outer tetragons is the numeric parameter of planarity deviation.

**The twist angle between two adjacent quartets in a quadruplex**

 In order to determine the quadruplex twist angle, the angle between the two vectors was measured. The first vector joined the C1’ atoms of two adjacent nucleotides in a quartet, while the second vector joined the C1’ atoms of the corresponding nucleotides in the adjacent quartet (Fig. 4). 

**The out-of-plane deviations of the quartet**

 The distance between the centers of mass of two tetragons (an original parameter) was proposed as a method for measuring the degree of disturbance of symmetry and planarity for an individual quartet. The first tetragon is formed by four N9 atoms of guanine in the quartet; the second tetragon is formed by four O6 atoms of the same guanines (Fig. 4). If the quartet is symmetrical and all guanines form hydrogen bonds with each other, this parameter fixes the planarity of the quartet. If the hydrogen bonds break, the symmetry of the quartet is disturbed; the parameter fixes the degree of quartet distortion, which includes deviations in both symmetry and planarity of a quartet. 

**The histogram of distribution of the quadruplex twist angles and out-of-plane deviations of the quartet**

 For each of the eight groups obtained, the values of the parameter were combined to plot a distribution histogram. The twist angle range was selected from 0 to 60°, the out-of-plane deviations were analyzed within a range from 0 to 2 Å, the ranges of angles and distances were partitioned into 15 intervals. The data were analyzed using Gnuplot software (http://www.gnuplot.info). 

##  RESULTS AND DISCUSSION 

**Parameters for description of geometry and conformational polymorphism of quadruplexes**

 Even a cursory analysis of the diversity of structure variants of the quartets detected in the PDB data bank leads to the conclusion that there is a necessity for systematization and development of universal parameters for the description of quartet structures and their polymorphism. Until now, no systematization attempts have been published. 

In this work, two parameters were selected as structural characteristics of the quadruplexes. The first parameter was the twist angle of the quadruplex; i.e., the angle between two adjacent quartets, which is described by the angle between two vectors passing through the C1’ atoms of two adjacent guanines (Fig. 4). This parameter has been widely used for the description of double-helix structures [31]. Previously it was demonstrated that the quadruplex twist angle represents the degree of tension of the oligonucleotide structure [32]. The second parameter was represented by an original parameter describing the planarity deviation of quartets; i.e., the distance between the centers of mass of two tetragons formed by the O6 and N9 atoms, respectively (Fig. 4). These two parameters make it possible to provide an adequate description of the conformational polymorphism and conformational mobility of the structure of quadruplex DNA.

**Four-stranded parallel quadruplexes**

 Composition of the group (ID PDB): 

**Fig. 5:**
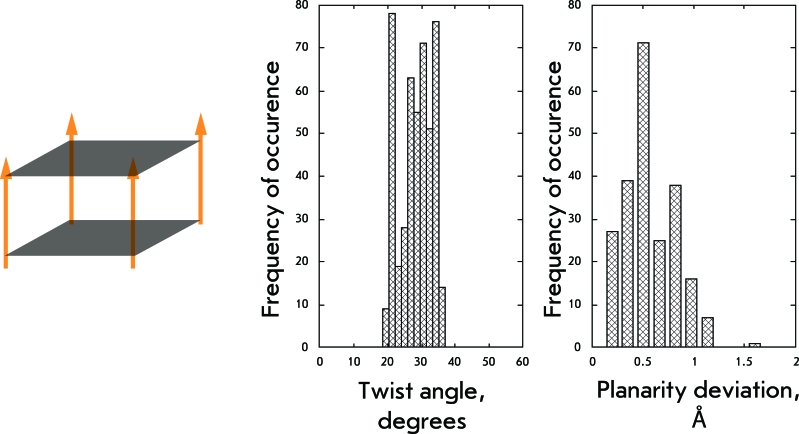
Structural organization and geometrical features of the first group of structures.

**Fig. 6:**
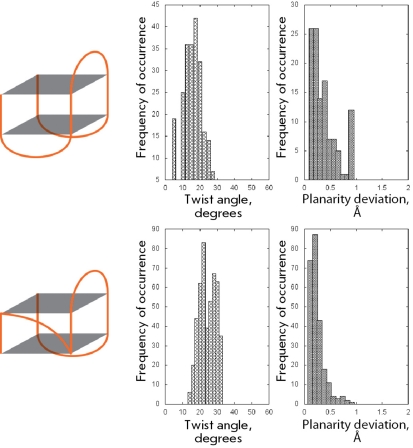
Structural organization and geometrical features of the second and third groups of structures.

• 1EVM [33], 1EVN [33], 1NP9 [34], 1NZM [35], 1O0K [36] – telomeric DNA (human);

• 139D [23, 37] – telomeric DNA ( *Tetrahymena* );

• 1EMQ [38] – telomeric DNA ( *Saccharomyces cerevisiae* );

• 1EVO [39] – fragment of SV40 viral genome.

 The group under consideration is the simplest variant of the arrangement of quadruplex structures. The twist angle in such structures may be regarded as the ideal twist angle, since the association of four strands imposes no structural restrictions at all, something not observed for the bimolecular and monomolecular quadruplexes. 

 Four-stranded parallel quadruplexes are characterized by a wide spectrum of twist angles (Fig. 5), with two ranges of preferred values: the narrow range corresponds to 21° and the more diffuse range lies within 27°–34°. In addition, the planarity of the quartets with this structure varies. In most cases, the quartets have a small out-of-plane deviation amounting to 0.5 Å; however, the maximum deviations are above 1 Å. The determined polydispersity of parameters illustrates the wide range of possibilities of the conformational polymorphism of four-stranded parallel quadruplexes without any structural restrictions for their formation upon intermolecular association. 

**Chair-type structure**

 Composition of the group: 

• 148D [40], 1C32 [41], 1C34 [41], 1C35 [41], 1C38 [41], 1QDF [42], 1QDH [42], 1RDE [43] – thrombin-binding DNA aptamer;

• 2KM3 [22] – telomeric repeat CTAGGG (human).

 The chair-type structure is a monomolecular quadruplex which is connected by three lateral loops. It is a sufficiently unique structure represented by only two molecules: a 15-mer thrombin-binding DNA aptamer (148D, 1C32, 1C34, 1C35, 1C38, 1QDF, 1QDH, 1RDE) and a 22-mer oligonucleotide, which is formed by the telomeric repeat CTAGGG (2KM3). Such structures are characterized by significantly smaller twist angles in comparison with those of the preceding group of “loopless” intermolecular four-stranded quadruplexes (Fig. 6). The mean value of quadruplex twist angles for the chair-type structures is equal to 15° with a deviation of ± 5°. The quartets with such structure are characterized by a high planarity; the jump in values within the range of 0.8–0.9 Å corresponds to the structures that can exist in high ionic strength solutions. The planarity results from the fact that the heterocyclic bases of the lateral loops form stacking interactions with the bases of the quartets, thereby limiting their out-of-plane deviations. 

**Monomolecular quadruplexes with (3+1) strand topology**

 Composition of the group: 

**Fig. 7:**
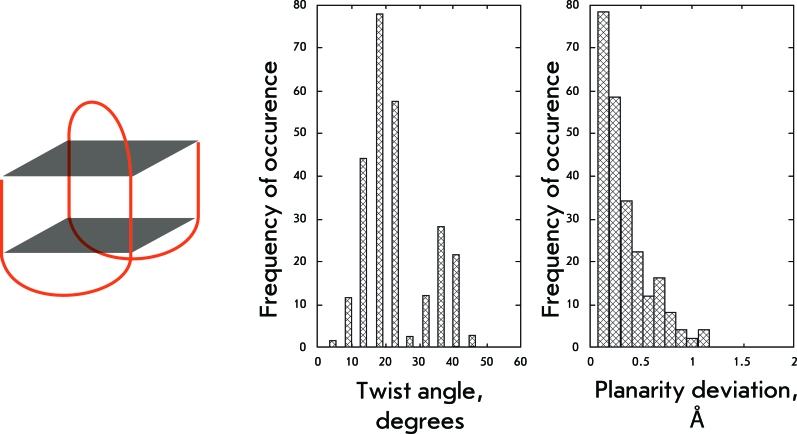
Structural organization and geometrical features of the fourth group of structures.

**Fig. 8:**
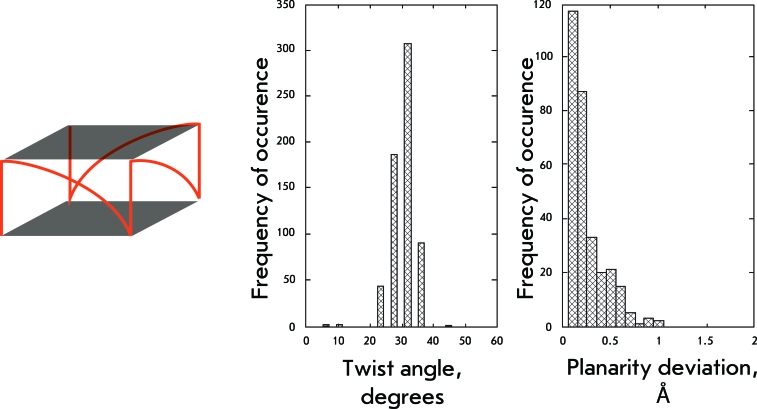
Structural organization and geometrical features of the fifth group of structures.

• 2JSK [44], 2JSQ [44], 186D [45], 2GKU [46], 2HY9 [47], 2JPZ [48], 2JSL [44], 2JSM [44] – telomeric DNA (human, *Tetrahymena* );

• 2F8U [49] – BCL2 promoter (human).

 These quadruplexes comprise two lateral loops and one propeller-type loop, which reverses the direction of the polynucleotide strand. This group differs from the preceding quadruplex type only by the existence of a propeller strand, which significantly affects the values of the twist angle (Fig. 6). An additional distribution with a maximum at 28° ± 4° emerges in the pattern of the angle distribution. Furthermore, the values of the twist angles within the range of 10°–20°, characteristic of quadruplexes with lateral loops, are shifted toward higher values by 2°–4°. Quadruplex quartets of this type are highly planar structures. Similar to the quartets in the previous case, the planarity is fixed by stacking interactions with heterocyclic bases of lateral loops. 

**Basket-type structure**

 Composition of the group: 

• 2KF8 [50], 2KKA [51], 2KOW [52], 143D [23], 230D [53], 201D [54] – telomeric DNA (human, *Oxytricha* ).

 The basket-type structures are represented by the monomolecular quadruplex connected by two lateral and one diagonal loops. As shown in the previous case, the substitution of a lateral loop by a diagonal one results in change in the character of the twist angle distribution and the emergence of two strongly pronounced shoulders with mean values of 18° ± 4° and 36° ± 4° (Fig. 7). Thus, a new set of structures, characterized by quadruplex twist angles higher than the maximum angles in loopless four-stranded parallel quadruplexes, emerging in the conformational landscape are conditioned by the emergence of a diagonal loop. The degree of planarity of basket-type quartet structures is lower than that of the earlier discussed structures with lateral loops, which is likely the result of a decrease in stacking interactions between the heterocyclic bases of the diagonal loop and the top quartet. 

**Monomolecular parallel quadruplexes with propeller loops**

 Composition of the group: 

• 1KF1 [24], 3CDM [55] – telomeric DNA (human);

• 1XAV [56], 2A5P [57], 1A5R [57] – c-MYC promoter (human);

• 2KQG [58], 2KQH [58], 2KYP [59] – c-kit oncogene promoter (human);

• 1MYQ [60] – synthetic oligonucleotide (GGA) _4_ ;

• 1Y8D [61] – aptamer-targeting HIV-1 integrase.

 These unusual structures represent intramolecular quadruplexes where all loops are of the propeller type and the polynucleotide strand changes its direction thrice. The presence of propeller loops strictly determines the quadruplex structure; the distribution of twist angles has a pronounced maximum at 31° ± 3° (Fig. 8). This value is similar to that determined for loopless four-stranded parallel quadruplexes. Quadruplexes of this type have planar quartets. It is not unlikely that propeller loops ensure the optimal geometry for quadruplexes with a rigid sugar-phosphate backbone. 

**Bimolecular quadruplexes with lateral loops**

 Composition of the group: 

• 1A8N [62], 1A8W [63] – tandem repeat GGGC;

• 1F3S [64] – synthetic oligonucleotide.

**Fig. 9:**
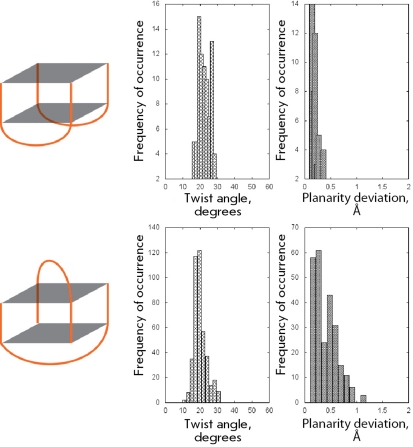
Structural organization and geometrical features of the sixth and seventh groups of structures.

 Unlike the monomolecular quadruplexes that are formed by intramolecular folding, bimolecular quadruplexes are formed upon dimerization of two self-folded polynucleotide strands which contain guanine blocks. The members of this quadruplex group are notable for the relatively long length of lateral loops (5–6 nucleotides). They can be regarded as an intermediate group between monomolecular quadruplexes with lateral loops and quadruplexes with diagonal loops. The values of the twist angles in these quadruplexes, lying in the region between two extrema belonging to the adjacent groups, argue for an intermediate position for this group as well (Figs. 6, 7, 9). It should be noted that the structures under consideration have a tendency to form 20° ± 1° and 27° twists. A higher statistical significance is required in order to draw firmer conclusions. The quartets in quadruplexes of this type appear to be highly planar due to the intense stacking interactions with the heterocyclic bases of the loops. 

**Bimolecular quadruplexes with diagonal loops**

 Composition of the group: 

**Fig. 10:**
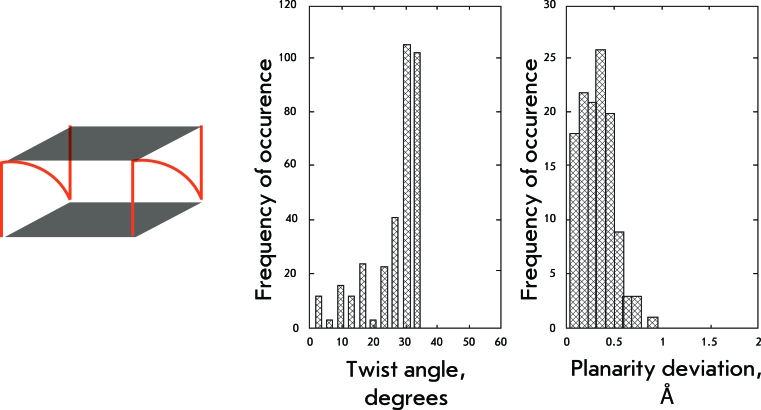
Structural organization and geometrical features of the eight group of structures.

• 156D [53, 65], 1JPQ [25], 1L1H [66], 1QDI [42], 1QDK [42], 3EM2 [67], 3EQW [67], 3ERU [67], 3ES0 [67], 3ET8 [67], 3EUM [67], 2AKG [68], 1K4X [53], 1JRN [25], 2HBN [69], 3EUI [67] – telomeric DNA ( *Oxytricha* );

• 2KAZ [70], 1U64 [71], 1LVS [72], 1FQP [73] – synthetic oligonucleotides.

 The molecules of this group form bimolecular quadruplexes with diagonal loops between the opposite angles of the quadruplex. Thus, these quadruplexes resemble the earlier described basket-type structures with two lateral loops substituted by a diagonal loop. In terms of the values of the twist angles, this substitution leads to the elimination of the shoulder with a maximum at 35°, retaining only one maximum at 19° ± 4° (Fig. 9). The distribution of the values of quartet planarity also resembles that of the basket-type structures. 

**Bimolecular parallel quadruplexes with propeller loops**

 Composition of the group: 

• 1K8P [24], 2HRI [74], 3CE5 [75] – telomeric DNA (human); 2KYO [59] – c-kit oncogene promoter (human);

• 1NYD [76], 1EEG [77], 1XCE [78] – synthetic oligonucleotides.

 For this type of quadruplexes, as well as for their monomolecular analog, propeller loops restrict the conformational polymorphism of the quadruplex: the distribution of the twist angle values has a maximum at 31° ± 3°; the quartets are planar (Fig. 10). 

**Classification of quadruplex structures**

 G-quadruplex structures are localized at the ends of telomeric regions of DNA and promoters of a number of oncogenes and cancer-associated genes. This fact makes the quadruplexes an attractive target for anti-cancer chemotherapy. The most preferable model of interaction between the anti-cancer agents and quadruplex DNA is based on stacking interactions with the quartets (terminal, or via intercalation), on one side, and interactions with the grooves, on the other side. Thus, thorough knowledge of the geometry of these elements and the factors that can influence them is a crucial part in a rational search for new efficient anti-cancer agents. 

 Nucleic acid quadruplexes are of interest not only as targets for anti-oncogenic drugs, but also as the structural core of aptamer-based therapeutic agents. In particular, this refers to aptamers targeting thrombin, HIV-1 integrase, the tumor marker – nucleolin protein which is involved in RNA processing. In order to optimize the stability and efficient self-assembling of these oligonucleotides, it is necessary to have knowledge pertaining to the nature of the forces able to have stabilizing or destabilizing effects on the aptamer molecule. 

 A wide topological and structural diversity conditioned by such variables as the number of G-quartets, the orientation, type, sequence and length of the loops is typical for quadruplexes;. For the description of this conformational ensemble, two parameters were selected: the twist angle between two adjacent quartets and the planarity of the quartets. 

 In our study it was shown that these parameters successfully characterize the most complex quadruplex structures. It should be noted that by the term “planarity” we refer to the generalization of two phenomena. When the quartet is symmetrical and all guanines form hydrogen bonds with each other, it is the planarity of the quartet that is fixed by this parameter. In the case of hydrogen bond breaking, the symmetry of the quartet is violated, and the parameter shows the degree of quartet distortion. 

 A four-stranded intermolecular parallel quadruplex is the simplest case, where only one of the specified parameters (the number of G-quartets) is realized. This variable determines the range in which these structures can exist without losing their integrity. This range is appreciably wide; in terms of the twist angle it stretches 19°–36°, with two regions of preferred values: 21° and 27°–34°. The motion of the terminal quartets in such structures is limited only by stacking interactions with the adjacent quartets and by coordination bindings with stabilizing cations if they exist in this case. Thus, G-quartets of these structures, in particular terminal ones, do not exhibit pronounced planarity. 

 Chair-type structures dramatically differ from the loopless four-stranded quadruplexes. The lateral loops of these structures restrict the geometry of quadruplexes; furthermore, they desrease the twist angles to values that are not characteristic of loopless quadruplexes. Whereas the minimum angles for loopless quadruplexes are equal to 19°, in contrast the structures containing only lateral loops have a range of preferred values that lies within 15° ± 5°. This points to the fact that considerable tension is introduced into the structure of the quadruplex by the lateral loops, primarily because of their small length. Meanwhile, on account of the location features evident from their name, loops of this type interact eagerly with the terminal quartets of quadruplexes at the terminal poles of G-quadruplexes via the formation of stacking interactions, which has a positive effect on the planarity of chair-type quartets. 

 It is of interest that the addition of diagonal or propeller loops to the chair-type structure has a considerable effect on the distribution pattern of twist angles. The preferred range of angle values shifts toward the higher values, typical of loopless parallel G-quadruplexes. In addition, a second shoulder emerges on the pattern, characterizing the region being adjacent to a loop other than the lateral one. 

In basket-type structures the strength of the tension induced by the lateral loops located at one pole of the molecule is clearly visible. The moment of these forces turns the molecule region, which is adjacent to the diagonal loop, into a range of twist angles of 36° ± 4°. That is more than the required norm for loopless parallel quadruplexes; however, the diagonal loop compensates for this abnormality through its length and elasticity. It should be noted that these values of twist angles are not typical of structures with diagonal loops only. The quadruplex geometry is rather rigidly restricted by angle values of 19° ± 4° in these structures, which is still closer to values that are reasonable for loopless quadruplexes. Thus, it could be concluded that the tensions induced by the lateral loops are stronger than those induced by the diagonal loops. It is noteworthy that the *syn/anti* -conformation pattern, which has been frequently used for the description of quadruplexes, is represented by the largest number of combinations in the group of bimolecular quadruplexes with diagonal loops. Conversely, this group exhibits the smallest dispersion of quadruplex twist angles. This observation allows one to assume that the loop type is the dominant influence factor on the quadruplex structure.

 The propeller loops determine the geometry of a quadruplex in the strictest manner. The values of twist angles fluctuate within 31° ± 3° both for monomolecular and bimolecular tetraplexes only with propeller loops. Even the introduction of lateral loops into such structures has a weak effect on the twisting of the regions adjacent to the propeller loops; it is characterized by a range of 28° ± 4°. These parameters agree with the second range of preferred values for the loopless quadruplexes (27°–34°). It seems likely that these twist angles correspond to the optimum geometry of a G-quartet, since the tetrads of structures with propeller loops are notable for high planarity, unlike quadruplexes with the diagonal loops. 

##  CONCLUSIONS 

 In this work, we have investigated all known G-quadruplex structures. These structures can be divided into groups that are not only limited to the basis of their topology; indeed, many groups are represented by sequences of common or related origin. It should be noted that the final discussion did not encompass all of the structures, since some of them are characterized by a topology too specific for the creation of representative samples. We have proposed two parameters to be used for the description of the geometry of quadruplexes: the twist angle of the tetraplex and the G-tetrad planarity. We demonstrated that the loops connecting the G-strand blocks are the major source of tension in the quadruplex structure. Lateral loops result in the strongest alteration of the geometry of G-quadruplexes; however, their influence is balanced by the introduction of diagonal and propeller loops into the structure. The diagonal loops provide the strict determination of the quadruplex structure, as well: however, the tensions induced by them are not as high as in the case with lateral loops. Propeller loops are characterized by an optimum quadruplex geometry. 

## References

[1] Gellert M., Lipsett M., Davies D. (1962). Proc. Natl. Acad. Sci. USA..

[2] Arnott S., Chandrasekaran R., Marttila C.M. (1974). Biochem. J..

[3] Zimmerman S. (1976). J. Mol. Biol..

[4] Simonsson T. (2001). Biol. Chem..

[5] De Lange T. (2005). Cold Spring Harb. Symp. Quant. Biol..

[6] Wright W.E., Tesmer V.M., Huffman K.E., Levene S.D., Shay J.W. (1997). Genes Dev..

[7] Lei M., Podell E.R., Cech T.R. (2004). Nat. Struct. Mol. Biol..

[8] Sen D., Gilbert W. (1988). Nature..

[9] Sundquist W.I., Klug A. (1989). Nature..

[10] Gilbert D.E., Feigon J. (1999). Curr. Opin. Struct. Biol..

[11] Cogoi S., Xodo L.E. (2006). Nucleic Acids Res..

[12] Rankin S., Reszka A.P., Huppert J., Zloh M., Parkinson G.N., Todd A.K., Ladame S., Balasubramanian S., Neidle S. (2005). J. Am. Chem. Soc..

[13] Dexheimer T.S., Sun D., Hurley L.H. (2006). J. Am. Chem. Soc..

[14] Siddiqui-Jain A., Grand C.L., Bearss D.J., Hurley L.H. (2002). Proc. Natl. Acad. Sci. USA..

[15] Hurley L.H., von Hoff D.D., Siddiqui-Jain A., Yang D. (2006). Semin. Oncol..

[16] Nimjee S.M., Rusconi C.P., Sullenger B.A. (2005). Annu. Rev. Med..

[17] Shamah S.M., Healy J.M., Cload S.T. (2008). Acc. Chem. Res..

[18] Bock L.C., Griffin L.C., Latham J.A., Vermaas E.H., Toole J.J. (1992). Nature..

[19] Jing N., Zhu Q., Yuan P., Li Y., Mao L., Tweardy D.J. (2006). Mol. Cancer Ther..

[20] McMicken H.W., Bates P.J., Chen Y. (2003). Cancer Gene Ther..

[21] Jayapal P., Mayer G., Heckel A., Wennmohs F. (2009). J. Struct. Biol..

[22] Lim K.W., Alberti P., Guédin A., Lacroix L., Riou J., Royle N.J., Mergny J., Phan A.T. (2009). Nucl. Acids Res..

[23] Wang Y., Patel D. (1993). Structure..

[24] Parkinson G.N., Lee M.P.H., Neidle S. (2002). Nature..

[25] Haider S., Parkinson G.N., Neidle S. (2002). J. Mol. Biol..

[26] Horvath M.P., Schultz S.C. (2001). J. Mol. Biol..

[27] Phillips K., Dauter Z., Murchie A., Lilley D., Luisi B. (1997). J. Mol. Biol..

[28] Hud N.V., Smith F.W., Anet F.A., Feigon J. (1996). Biochemistry..

[29] Kankia B.I., Marky L.A. (2001). J. Am. Chem. Soc..

[30] Deng J., Xiong Y., Sundaralingam M. (2001). Proc. Natl. Acad. Sci. USA..

[31] el Hassan M.A., Calladine C.R. (1995). J. Mol. Biol..

[32] Reshetnikov R., Golovin A., Spiridonova V., Kopylov A., Šponer J. (2010). J. Chem. Theory Comput..

[33] Patel P.K., Koti A.S., Hosur R.V. (1999). Nucl. Acids Res..

[34] Gavathiotis E., Searle M.S. (2003). Org. Biomol. Chem..

[35] Gavathiotis E., Heald R.A., Stevens M.F.G., Searle M.S. (2003). J. Mol. Biol..

[36] Clark G.R., Pytel P.D., Squire C.J., Neidle S. (2003). J. Am. Chem. Soc..

[37] Wang Y., Patel D. (1992). Biochemistry..

[38] Patel P.K., Hosur R.V. (1999). Nucleic Acids Res..

[39] Patel P.K., Bhavesh N.S., Hosur R.V. (2000). Biochem. Biophys. Res. Commun..

[40] Schultze P., Macaya R.F., Feigon J. (1994). J. Mol. Biol..

[41] Marathias V.M., Bolton P.H. (2000). Nucleic Acids Res..

[42] Marathias V.M., Wang K.Y., Kumar S., Pham T.Q., Swaminathan S., Bolton P.H. (1996). J. Mol. Biol..

[43] Mao X., Marky L.A., Gmeiner W.H. (2004). J. Biomol. Struct. Dyn..

[44] Phan A.T., Kuryavyi V., Luu K.N., Patel D.J. (2007). Nucleic Acids Res..

[45] Wang Y., Patel D.J. (1994). Structure..

[46] Luu K.N., Phan A.T., Kuryavyi V., Lacroix L., Patel D.J. (2006). J. Am. Chem. Soc..

[47] Dai J., Punchihewa C., Ambrus A., Chen D., Jones R.A., Yang D. (2007). Nucl. Acids Res..

[48] Dai J., Carver M., Punchihewa C., Jones R.A., Yang D. (2007). Nucl. Acids Res..

[49] Dai J., Chen D., Jones R.A., Hurley L.H., Yang D. (2006). Nucl. Acids Res..

[50] Lim K.W., Amrane S., Bouaziz S., Xu W., Mu Y., Patel D.J., Luu K.N., Phan A.T. (2009). J. Am. Chem. Soc..

[51] Zhang Z., Dai J., Veliath E., Jones R.A., Yang D. (2010). Nucl. Acids Res..

[52] Hu L., Lim K.W., Bouaziz S., Phan A.T. (2009). J. Am. Chem. Soc..

[53] Smith F., Feigon J. (1992). Nature..

[54] Wang Y., Patel D.J. (1995). J. Mol. Biol..

[55] Parkinson G.N., Cuenca F., Neidle S. (2008). J. Mol. Biol..

[56] Ambrus A., Chen D., Dai J., Jones R.A., Yang D. (2005). Biochemistry..

[57] Phan A.T., Kuryavyi V., Gaw H.Y., Patel D.J. (2005). Nat. Chem. Biol..

[58] Hsu S.D., Varnai P., Bugaut A., Reszka A.P., Neidle S., Balasu­bramanian S. (2009). J. Am. Chem. Soc..

[59] Kuryavyi V., Phan A.T., Patel D.J. (2010). Nucl. Acids Res..

[60] Matsugami A., Ouhashi K., Kanagawa M., Liu H., Kanagawa S., Uesugi S., Katahira M. (2001). J. Mol. Biol..

[61] Phan A.T., Kuryavyi V., Ma J., Faure A., Andréola M., Patel D.J. (2005). Proc. Natl. Acad. Sci. USA..

[62] Kettani A., Bouaziz S., Gorin A., Zhao H., Jones R.A., Patel D.J. (1998). J. Mol. Biol..

[63] Bouaziz S., Kettani A., Patel D.J. (1998). J. Mol. Biol..

[64] Kettani A., Basu G., Gorin A., Majumdar A., Skripkin E., Patel D.J. (2000). J. Mol. Biol..

[65] Smith F., Feigon J. (1993). Biochemistry..

[66] Haider S.M., Parkinson G.N., Neidle S. (2003). J. Mol. Biol..

[67] Campbell N.H., Patel M., Tofa A.B., Ghosh R., Parkinson G.N., Neidle S. (2009). Biochemistry..

[68] Gill M.L., Strobel S.A., Loria J.P. (2005). J. Am. Chem. Soc..

[69] Gill M.L., Strobel S.A., Loria J.P. (2006). Nucleic Acids Res..

[70] Balkwill G.D., Garner T.P., Williams H.E.L., Searle M.S. (2009). J. Mol. Biol..

[71] Sket P., Crnugelj M., Plavec J. (2004). Bioorg. Med. Chem..

[72] Crnugelj M., Hud N.V., Plavec J. (2002). J. Mol. Biol..

[73] Keniry M.A., Strahan G.D., Owen E.A., Shafer R.H. (1995). Eur. J. Biochem..

[74] Parkinson G.N., Ghosh R., Neidle S. (2007). Biochemistry..

[75] Campbell N.H., Parkinson G.N., Reszka A.P., Neidle S. (2008). J. Am. Chem. Soc..

[76] Webba da Silva M. (2003). Biochemistry..

[77] Kettani A., Gorin A., Majumdar A., Hermann T., Skripkin E., Zhao H., Jones R., Patel D.J. (2000). J. Mol. Biol..

[78] Webba da Silva M. (2005). Biochemistry..

